# Direct resection is a safe and effective strategy to control seizures in patients with hypothalamic hamartoma

**DOI:** 10.1038/s41598-024-63480-3

**Published:** 2024-06-06

**Authors:** Yunwei Ou, Jingzhe Yuan, Chunde Li

**Affiliations:** 1 Department of Neurosurgery, Beijing Tiantan Hospital, Capital Medical University, Beijing, China; 2 The Institute of Artificial Intelligence, Hefei Comprehensive National Science Center, Hefei, Anhui China; 3 Beijing Advanced Innovation Center for Big Data-Based Precision Medicine, Beihang University, Beijing, China; 4 China National Clinical Research Center for Neurological Diseases, Beijing, China

**Keywords:** Gelastic seizure-free, Hypothalamic hamartoma, Long-term, Overall seizure-free, Surgery, Medical research, Surgical oncology

## Abstract

Achieving favorable seizure outcomes is challenging in patients with seizures resulting from hypothalamic hamartoma. Although minimally invasive and non-invasive surgical procedures are used to treat this population, these procedures have limitations. Therefore, we analyzed the outcomes of patients with hypothalamic hamartoma following direct resection. We included 159 patients with hypothalamic hamartoma who underwent direct resection using the transcallosal interforniceal approach between 2011 and 2018. The relationships between clinical parameters and seizure outcomes were analyzed. In total, 55.3% achieved gross total resection and 25.2% underwent near-total resection. Of all patients, 79.2% were overall seizure-free at one year, but this number dropped to 77.0% at more than five years. Moreover, 88.4% (129/146) reached gelastic seizure (GS)-free status at one year and this number increased to 89.0% (97/109) at more than five years. Seventy-one patients took antiseizure medication (ASM) long-term, 68 took it for one year, and 11 took it for one-half year. The duration of ASM consumption (*p* < 0.001) and extent of hypothalamic hamartoma resection (*p* = 0.016) were significant independent predictors of long-term overall seizure-free survival, while the duration of ASM consumption (*p* = 0.011) and extent of hypothalamic hamartoma resection (*p* = 0.026) were significant independent predictors of long-term GS-free survival. Most patients' behavior, school performance, and intelligence were not affected after surgery. Direct resection is effective and safe strategy for patients with hypothalamic hamartomas. Hypothalamic hamartomas should be removed as completely as possible, and patients should take ASM long-term following surgery to reach long-term overall seizure-free or GS-free status.

## Introduction

Hypothalamic hamartomas are congenital developmental malformations consisting of neuronal and glial tissues in the tuber cinereum and mammillary bodies^1^. Two prototypical clinical phenotypes of hypothalamic hamartoma have been described^2,3^: one is located in the tuber cinereum and presents with precocious puberty and the other is located in mammillary bodies and is usually accompanied by epilepsy that frequently presents with drug-resistant gelastic seizures (GSs)^4^. Aside from GSs and other seizures, secondarily generalized seizures can also occur in patients with hypothalamic hamartoma with cognitive impairment and severe behavioral disturbances^3,4^. Minimally invasive or non-invasive surgical procedures (e.g., magnetic resonance imaging‐guided laser interstitial thermal therapy [MRg‐LITT], stereotactic radiofrequency thermocoagulation [SRT], and gamma knife radiosurgery) can be used to treat treating patients with hypothalamic hamartoma; however, they have limitations and can lead to postoperative complications. In addition, several neurosurgery centers do not have access to this type of equipment, particularly MRg‐LITT. Therefore, several surgical approaches, including transcallosal, interforniceal, and pterional approaches, have been established to treat hypothalamic hamartoma; these approaches can also achieve excellent seizure control and improve behavioral and neuropsychiatric symptoms^5–7^. However, little is known about the factors that predict better long-term symptom control after invasive surgeries. Therefore, this study aimed to analyze the clinical factors influencing the long-term control of seizures after direct resection surgery.

## Methods

A total of 159 patients < of 19 years old with refractory epilepsy due to hypothalamic hamartoma, who were treated at our hospital between 2011 and 2018, were included. All patients underwent surgical resection for the first time by a single experienced physician using the transcallosal interforniceal approach. The intraoperative navigation system was used to locate the tumor in patients with Delalande’s type I during surgery, and patients with hypothalamic hamartoma who were not treated surgically were excluded.

Using medical records, we reviewed each patient’s age, sex, endocrinological data, manifestations of precocious puberty, lesion type and size, history of intellectual disability, epilepsy history, duration of epilepsy, seizure onset, seizure frequency and type, and age at diagnosis. Data on scalp electroencephalography (EEG) or video‐EEG recordings, the number and types of anti-seizure medications (ASM) used, duration of ASM use, and extent of resection, were collected. Surgical complications, including short-term (central diabetes insipidus, electrolyte disturbances, hyperthermia, and endocrine deficits) and long-term (weight gain) complications, were recorded. We also recorded the body mass index (BMI) and the z-score of BMI was calculated according to Chinese data^8,9^. Weight gain was defined according to the Chinese standard^10^. Duration of ASM use was defined as the period of continuous ASM use.

The extent of surgical resection was classified into four categories based on the comparison of preoperative and postoperative magnetic resonance imaging (MRI): gross total resection (GTR; complete resection), near-total resection (NTR; removed ≥ 95% of the lesion), subtotal resection (STR; removed 50%–95% of the lesion), and partial resection (PR; removed < 50% of the lesion). Two independent neurosurgeons followed-up with the patients every 3 months via telephone and asked them about any postoperative seizures and functional outcomes. The results were summarized and statistically analyzed at the end of the project. The two neurosurgeons were independent follow-up personnel in our department who did not have access to the final results of the project and were only responsible for the follow-up. Upon discharge, we would provide training to the patient's family members so that they can proficiently administer the cognitive function scale and intelligence scale at home. This would enable them to monitor and record any changes in the patient's cognitive function, intellectual abilities. They would also be trained to observe and record the patient's behavioral, emotional, learning ability, and seizure changes (including various forms of seizures). During telephone follow-up sessions, we would document neurocognitive recovery progress, intelligence test results, behavior patterns, emotional states, and seizure records. All these findings will be reviewed by a physician during the patient's outpatient reevaluation at six months to one year post-surgery. “Short-term” was defined as the length of the patient's hospital stay to six months after surgery, while “long-term” was defined as the patient’s entire postoperative follow-up period. Short-term surgical complications included central diabetes insipidus, electrolyte disturbances, endocrine deficits, central nervous system (CNS) infection, and hyperthermia. Central diabetes insipidus was defined as a urine volume exceeding 2 L/m^2^–/24 h or > 150 ml/kg/24 h at birth, > 100–110 ml/kg/24 h until the age of 2 years old and > 40–50 ml/kg/24 h in older children and adults^11^. Electrolyte disorders were classified as mild hyponatremia (Na^+^ = 120–135 mmol/L), moderate hyponatremia (Na^+^ = 115–120 mmol/L), and severe hyponatremia (Na^+^  < 115 mmol/L). After discharge, all patients were required to have their blood electrolyte levels tested daily in the outpatient department for two weeks. If their electrolyte levels were abnormal, they would be treated in the outpatient department. Otherwise, their blood electrolyte levels would be checked again in the outpatient department at one month, two months and three months after surgery. Hyperthermia was defined as a body temperature between 39.1 and 41 °C. All patients would undergo routine cerebrospinal fluid (CSF) examination by lumbar piercings after surgery. We defined CNS infection based on patient's symptoms, signs, routine CSF testing and culture results^12^. The endocrine deficits of all patients in this study were manageable and could be relieved by substituting of hormone medications.

Behavioral assessments were mainly performed in patients older than two years. Information pertaining to preoperative behavior was obtained from the clinical records, whereas postoperative behavior was assessed according to the parents’ written reports. Behavioral outcomes included hyperactivity, irritability, aggressiveness, autism, and temper tantrums. The results of parents’ and teachers’ written reports were used to assess school performance both pre- and postoperatively, while the Wechsler Intelligence Scale for Children–Revised was used to evaluate general intelligence in patients older than six years of age. The parents’ written reports mainly recorded their children's homework completion. The teachers’ written reports mainly recorded the children's daily learning at regular schools and focused on significant learning difficulties and the need for special schooling. Postoperative behavior, school performance, and general intelligence were assessed from one year or more follow-up after the operation. Informed consent was obtained from all subjects or their legal guardians., and ethical approval was obtained from the Institutional Research Ethics Committee of Beijing Tiantan Hospital (KY2022-067-01). All methods were carried out in accordance with relevant guidelines and regulations. The datasets for the current study are available from the corresponding author upon request.

### Statistical analyses

We examined the normality of the clinical parameters prior to performing statistical analyses; non-normally distributed clinical parameters were analyzed using non-parametric tests. We analyzed differences in clinical parameters using the *t*-test or Chi-squared test and used multiple logistic regression analysis to assess the relationships between clinical parameters and seizure outcomes. Statistical analyses were performed using SPSS software version 17.0.0, and *p* < 0.05 was considered to indicate statistical significance.

### Ethical approval

This study was approved by the Institutional Research Ethics Committee of Beijing Tiantan Hospital, Capital Medical University, China (No. KY 2022-067-01).

## Results

A total of 103 men and 56 women had hypothalamic hamartomas. The average length of hospital stay was 10.3 ± 5.2 days (range, 8.0–16.0 days), and the postoperative follow-up period was 7.0 ± 2.1 years (range, 3.7–11.3 years) with a 100.0% follow-up rate. The onset time for epilepsy ranged from the first month of life to 13 years of age (1.9 ± 1.0 years), and age at surgery ranged from 8 months to 19 years (6.9 ± 4.5 years). The average age at the interval between the onset of epilepsy and surgery was 5.0 ± 3.9 years (range, 0–16.9 years). Initial symptoms included GS, precocious puberty, and others; 86.8% presented with GS as an initial symptom and 6.9% presented with precocious puberty. Among patients with GS, 34.9% (51/146) displayed GS as the only symptom, 51.4% (75/146) of patients manifested GS combined with other seizures, and 13.7% (20/146) of patients displayed GS combined with precocious puberty. Of the patients with precocious puberty as the initial symptom, seven showed precocious puberty in combination with GS. The interval time from precocious puberty to other seizures was 14.2 ± 17.2 months. According to the mean maximum transverse diameter of the lesion on the T1 axis of MRI (17.0 mm), we divided the patients into ≤ 17 mm group and > 17 mm group. We found that 54.7% patients were in the ≤ 17-mm group and 45.3% were in the > 17-mm group. Moreover, 50.3% patients were classified as type III according to Delalande’s classification^13^, while 31.4% of patients were type II, 15.7% of patients were type IV, and 2.5% of patients were type I.

No patients died after surgery. In terms of the short-term surgical complications, 9.4% developed central diabetes insipidus (including 13 cases of mild diabetes insipidus, one case of moderate diabetes insipidus, and one severe case of diabetes insipidus), 49.7% had electrolyte disturbances (including 65 with mild hyponatremia, 10 with moderate hyponatremia, and four with severe hyponatremia), 22.6% had endocrinologic deficits and 4.4% of patients developed hyperthermia. We found that 8.8% of patients had mild and manageable operation-related CNS infection, characterized by new fever and increased CSF white blood cells on CSF examination, and none had a positive CSF culture. Therefore, we treated them by regular lumbar piercings to release CSF and promoted CSF circulation without antimicrobial therapy. If there was a sustained increase of CSF white blood cell count or positive CSF culture or the patient had new symptoms such as new headache, nausea, lethargy and so on, we would use antimicrobial therapy. We found that all patients with CNS infections were able to recover within a week by releasing CSF through daily lumbar piercings. Five patients with central diabetes insipidus recovered before hospital discharge. The remaining patients discharged from the hospital after recovery included 39 with electrolyte disturbances, one with endocrine deficits, 14 with infections, and five with hyperthermia. All patients with short-term surgical complications recovered within three months after surgery. Of all patients, 5.7% experienced weight gain (Table 1).

**Table 1:** Clinical parameters of 159 patients with hypothalamic hamartoma.

Clinical parameters	Patients n (%)
Sex	
Male	103 (64.8)
Female	56 (35.2)
Onset time of epilepsy (years)	1.9 ± 1.0
Age at surgery (years)	6.9 ± 4.5
Interval between onset of epilepsy and surgery (years)	5.0 ± 3.9
Initial symptom	
GS	138 (86.8)
PP	11 (6.9)
Other epilepsies	10 (6.3)
Symptoms	
GS	51 (32.1)
GS + Other epilepsies	75 (47.2)
Other epilepsies	11 (6.9)
GS + PP	20 (12.6)
PP + Other epilepsies	2 (1.3)
Lesion diameter	
Maximum (mm)	17.0 ± 7.7
≤ 17 mm	87 (54.7)
> 17 mm	72 (45.3)
Delalande’s classification	
I	4 (2.5)
II	50 (31.4)
III	80 (50.3)
IV	25 (15.7)
Surgical complications	
Short-term	
Central diabetes insipidus,	15 (9.4)
Electrolyte disturbances,	79 (49.7)
Endocrinologic deficits	36 (22.6)
Infection	14 (8.8)
Hyperthermia	7 (4.4)
Long-term	
Weight gain	9 (5.7)

*GS* gelastic seizure, *PP* precocious puberty.

In total, 85.1% (74/87) of patients achieved GTR in the ≤ 17-mm group, while 19.4% (14/72) achieved GTR in the > 17-mm group (*p* < 0.001). If patients could not reach GTR, hypothalamic hamartoma lesions were disconnected from the adjacent hypothalamus and mammillary bodies during surgery. All patients with type II 50.0% with type I underwent GTR. Of the patients, 37.5% with type III achieved NTR and 48% with type IV achieved STR (Table 2).

**Table 2:** The extent of resection of lesion in 159 patients with hypothalamic hamartoma.

	Extent of resection n (%)				*p*
	GTR	NTR	STR	PR	
Lesion diameter					< 0.001
≤ 17 mm	74 (85.1)	10 (11.5)	3 (3.4)	0 (0.0)	
> 17 mm	14 (19.4)	30 (41.7)	27 (37.5)	1 (1.4)	
Delalande’s classification					< 0.001
I	2 (50.0)	1 (25.0)	1 (25.0)	0 (0.0)	
II	50(100.0)	0 (0.0)	0 (0.0)	0 (0.0)	
III	30 (37.5)	32 (40.0)	17 (21.3)	1 (1.3)	
IV	6 (24.0)	7 (28.0)	12 (48.0)	0 (0.0)	
Total	88 (55.3)	40 (25.2)	30 (18.9)	1 (0.6)	

*GTR* gross total resection, *NTR* near-total resection, *STR* subtotal resection, *PR* partial resection.

The side effects that persisted for more than one year after surgery were almost irreversible, especially abnormal behavior, poor school performance, and decreased intelligence. We found that 49 patients with behavioral abnormalities displayed improvement in behavior after surgery. Among these, 30 patients stopped experiencing temper tantrums, 12 patients experienced a shift from severe hyperactivity to mild hyperactivity, and seven patients’ aggressiveness resolved. Twenty-five patients who did not have behavioral problems before surgery developed certain abnormalities after surgery, including four patients with hyperactivity (two mild, one moderate, and one severe), six with irritability (two mild, three moderate, and one severe), three with aggressiveness (two mild and one moderate), two with mild autism, and ten with temper tantrums (six mild, three moderate, and one severe). Fifty-seven patients attended school before surgery and 102 patients attended school after surgery. Among those who had gone to school before surgery, 12.3% (7/57) appeared to have worse school functioning and poorer attention and memory compared with pre-surgery parameters, while 14.0% (8/57) displayed improved school performance after surgery. Preoperative and postoperative intelligence were measured in 82 patients aged < 6 years; intelligence improved in 14.6% (12/82) of patients after surgery compared to pre-operation, while 6.1% (5/82) of patients deteriorated (Table 3).

**Table 3:** The outcome of behavior, school performance and general intelligence for patients with hypothalamic hamartoma.

	Outcome	
	Deteriorated n (%)	Improvement n (%)
Behavioral outcome (n = 139)	25 (18.0)	49 (35.3)
School performance (n = 57)	7 (12.3)	8 (14.0)
Intelligence (n = 82)	5 (6.1)	12 (14.6)

All patients took ASM after surgery, which included levetiracetam (92/159), sodium valproate or depakine (14/159), oxcarbazepine (56/159), magnesium valproate (2/159), and lamotrigine (2/159). Patients underwent routine EEGs to monitor ASM use during the follow-up period. One patient used three types of ASMs, four self-administered two types of ASMs, and the others showed controlled seizures with one type of ASM. One patient stopped taking oxcarbazepine after five years, whereas another patient took depakine for three years. Seven patients took levetiracetam or oxcarbazepine for two years, 68 patients took ASM for one year, and 11 for one-half year. The others took ASM long term and did not discontinue it. Seizure outcomes of all patients were analyzed after surgery. We assessed the extent of remission of all seizure symptoms after surgery as short-term (0–6 months) and long-term outcomes (≥ 1 year) (Table 4). We found that 83.0% (132/159) of patients were completely overall seizure-free (Engel I) and 17.0% (27/159) were Engel II + III in the short term. The proportion of patients who were overall seizure-free (Engel I) decreased to 79.2% (126/159) at one year after surgery (*p* = 0.005). However, the rate of patients who were postoperative overall seizure-free postoperatively (Engel I) gradually decreased and dropped to 77.0% (87/113) at more than five years after surgery. Finally, we focused on the extent of remission of GS after surgery and found that 82.2% (120/146) of patients with GS achieved complete control (Engel I) in the short term, while 88.4% (129/146) reached Engle I classification at one year after surgery (*p* < 0.001). Interestingly, the rate of complete GS-free (Engel I) gradually increased to 89.0% (97/109) at more than five years after surgery.

**Table 4:** The short-term and long-term seizure outcome for patients with hypothalamic hamartoma.

Seizure outcome	Overall seizure-free n (%) (n = 159)							Gelastic seizure -free n (%) (n = 146)						
	0–6 MOS n (%)	Long-term					*p*	0–6 MOS n (%)	Long-term					*p*
		1y n (%) (n = 159)	2y n (%) (n = 159)	3y n (%) (n = 159)	4y n (%) (n = 145)	≥ 5y n (%) (n = 113)			1y n (%) (n = 146)	2y n (%) (n = 146)	3y n (%) (n = 146)	4y n (%) (n = 138)	≥ 5y n (%) (n = 109)	
Engel I	132 (83.0)	126 (79.2)	123 (77.4)	121 (76.1)	114 (78.6)	87 (77.0)	0.005	120 (82.2)	129 (88.4)	127 (87.0)	125 (85.6)	122 (88.4)	97 (89.0)	< 0.001
Engel II + III	27 (17.0)	33 (20.8)	36 (22.6)	38 (23.9)	31 (21.4)	26 (23.0)		26 (17.8)	17 (11.6)	19 (13.0)	21 (14.4)	16 (11.6)	12 (11.0)	

We further examined the clinical parameters that contributed to long-term overall seizure-free status via multiple logistic regression analyses. We divided patients into two groups based on whether GTR was achieved and found that the duration of ASM use (years) (*p* < 0.001) and extent of resection of hypothalamic hamartoma (*p* = 0.016) were significant independent predictors of long-term overall seizure-free status (Table 5). We also found that the duration of ASM (years) (*p* = 0.011) and extent of hypothalamic hamartoma resection (*p* = 0.026) were significant independent predictors of long-term GS-free status (Table 6).

**Table 5:** Multivariate logistic regression analyses of clinical parameters related to postoperative long-term overall seizure-free in 159 patients with hypothalamic hamartoma.

Clinical parameters	*P*_U_	B	Exp (B)	95% (C.I.)	*P*_M_
Gender	0.877				
Onset time of epilepsy (years)	0.445				
Age at surgery (years)	0.248				
Interval between onset of epilepsy and surgery (years)	0.641				
Initial symptom	0.283				
Gross total resection (Yes vs No)	< 0.001	0.860	2.363	1.174–4.753	0.016
Maximum diameter	< 0.001	− 0.018	0.982	0.896–1.076	0.695
duration of take ASM (years)	< 0.001	0.688	1.990	1.493–2.652	< 0.001
Delalande’s classification	0.003	0.681	1.976	0.776–5.031	0.153

ASM: antiseizure medication. *P*_U_: *P* value of Univariable logistic regression analyses; *P*_M_: P value of Multivariable logistic regression analyses.

**Table 6:** Multivariate logistic regression analyses of clinical parameters related to postoperative long-term overall GS-free in 146 patients with hypothalamic hamartoma.

Clinical parameters	*P*_U_	B	Exp (B)	95% (C.I.)	*P*_M_
Gender	0.221				
Onset time of epilepsy (years)	0.497				
Age at surgery (years)	0.713				
Interval between onset of epilepsy and surgery (years)	0.998				
Initial symptom	0.856				
Gross total resection (Yes vs No)	< 0.001	2.011	7.470	1.270–43.944	0.026
Maximum diameter	< 0.001	0.036	1.037	0.940–1.145	0.468
duration of take ASM (years)	0.014	0.3370	1.448	1.089–1.926	0.011
Delalande’s classification	0.024	0.034	1.034	0.351–3.048	0.951

ASM: antiseizure medication. *P*_U_: *P* value of Univariable logistic regression analyses; *P*_M_: *P* value of Multivariable logistic regression analyses.

## Discussion

In this study we investigated 159 patients with hypothalamic hamartomas who were surgically treated using the transcallosal interforniceal approach. We found that direct resection was an effective strategy as 77.0% of patients were overall seizure-free (Engel I) for five years and 89.0% were overall seizure-free for more than five years GS-free (Engel I). The extent of resection and duration of ASM use were identified as significant prognostic factors for long-term outcomes. Further, we found that direct and complete resection of hypothalamic hamartoma lesions can be helpful in improving behavior; however, their effects on learning and intelligence also need to be considered.

It is unclear whether complete resection of a hypothalamic hamartoma or anatomical disconnection from the hypothalamus and mammillary bodies is sufficient to achieve an overall seizure-free status. In accordance with the theory that disconnection of the hypothalamic hamartoma from the hypothalamus and mammillary bodies is sufficient to achieve overall seizure-free status, stereotactic radiofrequency thermocoagulation, gamma knife radiosurgery, endoscopic disconnection, and MRg‐LITT have been used to control the symptoms of hypothalamic hamartoma^14–17^. Munari et al. first discovered epileptiform discharges associated with GS that arose from hypothalamic hamartoma lesions^18^, and growing evidence has revealed the mechanism underlying hypothalamic hamartomas. First, many types of neurons show typical characteristics of depolarization and firing in response to GABA ligands, with pacemaker-like firing behavior in the hypothalamic hamartoma tissue^19,20^. MR imaging and ictal single-photon emission computed tomography studies show that hypothalamic hamartoma lesions initiate anatomic connections with the columns of the fornix, mammillary bodies, and mammillothalamic tracts. These results suggest that epileptiform discharges from hypothalamic hamartoma lesions can spread to temporal or frontal areas^21–24^. Further, a functional study revealed that hypothalamic hamartomas can cause other types of seizures in the temporal or frontal areas, such as secondary epileptogenesis^25,26^. GS can gradually develop into complex partial and/or generalized epilepsy without timely surgical intervention, and approximately 70% of patients with hypothalamic hamartomas exhibit other types of seizures before diagnosis^27^. In our study, among patients with GS as the initial symptom, 51.4% also had other seizures. Considering the mechanisms mentioned above and Morrell’s theory of the three stages of secondary epileptogenesis^28–30^, the hypothalamic hamartoma lesion should be completely removed as early as possible using surgical treatments. Ng et al., found that 54% (14/26) of hypothalamic hamartoma patients with refractory epilepsy treated with surgical resection (transcallosal interforniceal approach) were completely seizure free, while 35% (9/26) had at least a 90% improvement in the total seizure frequency^31^. In addition, Harvey et al. found that 52% (15/29) of patients with hypothalamic hamartoma became seizure-free following surgical resection (transcallosal interforniceal approach), and 24% (7/29) had > 90% reduction in seizure frequency^32^. These results suggest that hypothalamic hamartoma patients with refractory epilepsy may be safely and effectively treated with surgical resection using the transcallosal interforniceal approach^33^. Herein, we found that complete resection of hypothalamic hamartoma was an important factor in determining the overall seizure-free status, particularly with respect to being GS-free. The long-term outcomes of GS improved more significantly than those of other types of seizures (88.4% vs. 79.2%), which is consistent with previous reports^34,35^. We hypothesized that incomplete resection or disconnection of hypothalamic hamartoma lesions would not abrogate persistent epileptiform discharges from hypothalamic hamartoma lesions and only interrupt the hypothalamic hamartoma network for a period of time. Thereafter, hypothalamic hamartoma lesions would reconstruct a new network to spread epileptiform discharges to the surrounding areas. This hypothesis is supported by the fact that surgical treatment to strategically isolate hypothalamic hamartoma lesions allows some patients to be completely seizure-free, while others achieve partial remission with persistent electroclinical seizures^36,37^. In this study, we obtained high rates of postoperative overall seizure-free-(Engel I) (77.0%) and GS-free (Engel I) (89.0%) cases at more than five years after surgery.

MRg‐LITT, as a minimally invasive approach, has become the first-line treatment for hypothalamic hamartoma at some neurosurgery centers. However, different neurosurgery centers have different GS-free rates. For example, Yao et al. found that only 68.1% (32/47) of hypothalamic hamartoma patients reached Engel I GS-free status after one year by MRg‐LITT^38^, while Gadgil et al. found that 81.0% of patients were completely free of GS and 68.2% (15/22) of patients with secondary nongelastic epilepsy were free of additional seizures^39^. Further, Curry et al. found that 92% (33/36) of patients were seizure free at 1 year if resting state functional magnetic resonance imaging (rs-fMRI) was used during the LITT process, and only 47% (7/15) of patients achieved got Engel I seizure-free status when rs-fMRI was not used as a guide. In addition, 49.0% patients achieved overall seizure-free status at the last follow-up after stereotactic laser ablation (SLA) procedures when rs-fMRI was not used as guide^40^.

Patients with wide connection or Delalande IV hypothalamic hamartoma require multiple rounds of ablation by MRg‐LITT and displayed unfavorable outcomes, suggesting that patients with a larger hypothalamic hamartoma lesion or Delalande IV hypothalamic hamartoma are not suitable candidates for MRg‐LITT^38^. SRT is another minimally invasive surgical procedure that can achieve excellent seizure outcomes even in patients with very large hypothalamic hamartomas. Shirozu et al. found that 81.3% patients with very large hypothalamic hamartomas achieved freedom from GS after the final SRT procedure, but only 58.3% patients experienced freedom from non-GS^41^. These results suggest that a minimally invasive surgical procedure can lead to patients achieving a GS-free status; however, these patients may not achieve excellent outcomes in terms of non-GS freedom. The rate of postoperative overall seizure-free (Engel I) was 77.0% (87/113) and the rate of complete GS-free was 89.0% (97/109) at more than five years after surgery in our study.

In addition to seizure control, the effects on behavior and cognition after surgery are important factors for parents to consider when deciding whether their children should undergo surgery. Patients with hypothalamic hamartoma could benefit from surgery by improving intellectual functioning and behavioral and cognitive abnormalities^42–44^. Here, we analyzed the short-term postoperative complications and found that all patients recovered within three months after surgery. These transient complications also occurred after other less invasive or non-surgical treatments^45,46^. Long-term complications, such as memory impairment, weight gain, and worse school functioning, were reported following surgical interventions, minimally invasive surgery, and even non-surgical treatment. Significant memory impairments have also been reported in patients with hypothalamic hamartomas treated with stereotactic laser ablation^47^. Schulze-Bonhage et al. reported that 13.3% (2/15) of patients with hypothalamic hamartoma treated with interstitial radiosurgery had developed worsening of episodic memory without other serious impairments^48^. Adverse effects of MRg-LITT have also been reported, such as memory impairment and weight gain^38,49,50^. Yuan et al. reported that 2.1% of hypothalamic hamartoma patients had memory deficits after treatment with MRg-LITT, whereas 4.3% experienced weight gain as long-term complications^38^. We found that most patients' behaviors, school performance, and intelligence were not affected. Among patients whose behavior, school performance, or intelligence were affected by surgery, most showed improvement after surgery. Therefore, although minimally invasive or non-invasive surgical procedures can be effective for treating patients with hypothalamic hamartoma, they have limitations and can lead to postoperative complications. Overall, direct resection of hypothalamic hamartoma lesions using the transcallosal interforniceal approach is a highly effective and safe strategy in centers where minimally invasive or non-invasive surgical procedures are not available.

Administration of antiepileptic medications before interventional surgery is not recommended, as epilepsy arising from hypothalamic hamartoma lesions is known to be resistant to antiepileptic drugs, and taking medicine before surgery may be associated with worse cognitive function^51–54^. Although there is no evidence concerning the role of ASM in the natural history of hypothalamic hamartomas or any long-term benefit^55^, administration of this medication should be considered after surgery to prevent other seizure types. Intracerebral recordings (e.g., stereotactic EEG) are rarely used in the diagnosis of hypothalamic hamartoma, and the presentation of hypothalamic hamartoma on routine scalp EEG shows a wide range of distinct differences from normal EEG recordings to multiple interictal epileptiform features^56^, suggesting that EEG should not be included in the specific workup for the diagnosis of hypothalamic hamartoma and that it cannot be used to guide the termination of ASM use after surgery. We found that some patients who experienced controlled seizures for several years after the use of ASM developed drug-resistant seizures after discontinuing drug use. Therefore, the suitable time for stopping ASM use remains unclear. In this study, the recovery rate in the long-term GS-free group was higher than that in the short-term group. Multiple regression analyses revealed that the duration of ASM use was positively associated with long-term overall seizure-free and GS-free survival. These relationships suggest that the duration of administration of ASM is positively correlated with more favorable outcomes with respect to long-term overall seizure-free- or GS-free status. In addition, most patients exhibit controlled seizures when only one type of ASM is used after surgery. Type of ASM that patients took after surgery differed; however, we did not find a relationship between the type of ASM taken and hypothalamic hamartoma reappearance. This finding suggests that the type of ASM taken after surgery does not affect the outcomes of patients with hypothalamic hamartoma.

This study has some limitations. First, all patients underwent surgeries using the transcallosal interforniceal approach by an experienced physician, which ensured consistency of all surgical outcomes. However, for this reason, we were unable to compare the outcomes between other surgical approaches and physicians. Second, this was a retrospective study. In the future, a prospective study should be performed and more detailed neuropsychological and cognitive evaluations of factors such as verbal memory, speed, and executive function and long-term endocrine dysfunction in the pre- and post-operation periods should be conducted.

## Conclusions

Hypothalamic hamartomas are congenital lesions that can cause GSs and other types of epilepsy. Achieving long-term favorable outcomes in patients with epilepsy resulting from hypothalamic hamartoma remains challenging. It is important to select the appropriate treatment as the epilepsy can reoccur if it is not properly treated and managed. Here, we found that direct and complete resection of hypothalamic hamartoma lesions using the transcallosal interforniceal approach is a highly effective and safe strategy for achieving favorable results in patients with hypothalamic hamartoma when patients continue to take ASM long-term following surgery.

## Data Availability

The datasets used and/or analyzed during the current study are available from the corresponding author on reasonable request.
